# Cost-Effectiveness of the Manchester Approach to Identifying Lynch Syndrome in Women with Endometrial Cancer

**DOI:** 10.3390/jcm9061664

**Published:** 2020-06-01

**Authors:** Tristan M. Snowsill, Neil A. J. Ryan, Emma J. Crosbie

**Affiliations:** 1 Health Economics Group, University of Exeter Medical School, Exeter EX1 2LU, UK; 2 Division of Evolution and Genomic Medicine, University of Manchester, St Mary’s Hospital, Manchester M13 9WL, UK; neilryan@nhs.net; 3 Division of Cancer Sciences, Faculty of Biology, Medicine and Health, University of Manchester, St Mary’s Hospital, Manchester M13 9WL, UK; emma.crosbie@manchester.ac.uk; 4 Academic Centre for Women’s Health, University of Bristol, Bristol BS8 2PS, UK; 5 Division of Gynaecology, Manchester University NHS Foundation Trust, Manchester Academic Health Science Centre, Manchester M13 9WL, UK

**Keywords:** Lynch syndrome, endometrial cancer, reflex testing, cost-effectiveness analysis, decision analytic model

## Abstract

Lynch syndrome (LS) is a hereditary cancer syndrome responsible for 3% of all endometrial cancer and 5% in those aged under 70 years. It is unclear whether universal testing for LS in endometrial cancer patients would be cost-effective. The Manchester approach to identifying LS in endometrial cancer patients uses immunohistochemistry (IHC) to detect mismatch repair (MMR) deficiency, incorporates testing for *MLH1* promoter hypermethylation, and incorporates genetic testing for pathogenic MMR variants. We aimed to assess the cost-effectiveness of the Manchester approach on the basis of primary research data from clinical practice in Manchester. The Proportion of Endometrial Tumours Associated with Lynch Syndrome (PETALS) study informed estimates of diagnostic performances for a number of different strategies. A recent microcosting study was adapted and was used to estimate diagnostic costs. A Markov model was used to predict long-term costs and health outcomes (measured in quality-adjusted life years, QALYs) for individuals and their relatives. Bootstrapping and probabilistic sensitivity analysis were used to estimate the uncertainty in cost-effectiveness. The Manchester approach dominated other reflex testing strategies when considering diagnostic costs and Lynch syndrome cases identified. When considering long-term costs and QALYs the Manchester approach was the optimal strategy, costing £5459 per QALY gained (compared to thresholds of £20,000 to £30,000 per QALY commonly used in the National Health Service (NHS)). Cost-effectiveness is not an argument for restricting testing to younger patients or those with a strong family history. Universal testing for Lynch syndrome in endometrial cancer patients is expected to be cost-effective in the U.K. (NHS), and the Manchester approach is expected to be the optimal testing strategy.

## 1. Introduction

Lynch syndrome is an inherited cancer predisposition syndrome caused by pathogenic variants in the DNA mismatch repair (MMR) genes. It has incomplete penetrance, which is also dependent on which MMR gene is affected, but most individuals with Lynch syndrome face much higher risks of colorectal cancer, endometrial cancer, and ovarian cancer than the general population [1]. There are generally no signs or symptoms of Lynch syndrome other than developing cancer. An individual is generally regarded as having Lynch syndrome if they have a pathogenic MMR variant (path_MMR) regardless of whether they have been diagnosed with an associated cancer.

As individuals with Lynch syndrome are more likely to develop colorectal and endometrial cancer, the prevalence of Lynch syndrome is higher amongst those diagnosed with these cancers than in the general population. Further, it is possible to test colorectal and endometrial tumours for signs that Lynch syndrome may be implicated, as mismatch repair deficiency (dMMR) is associated with Lynch syndrome and can be detected through molecular testing for microsatellite instability (MSI) or through immunohistochemistry (IHC). Additionally, testing for hypermethylation of the *MLH1* promoter region is able to rule out some sporadic cancers that demonstrate dMMR. Reflex (automatic) testing of colorectal and endometrial cancer cases for possible Lynch syndrome, followed by referral for diagnostic genetic testing, is a proven strategy for identifying individuals (and their family members) with Lynch syndrome [2,3].

At present, testing for Lynch syndrome in endometrial cancer patients is not widespread in the U.K. The U.K. health service is dominated by the National Health Service (NHS), which provides healthcare mostly free of charge, paid for through general taxation (i.e., users are generally not required to purchase social or private medical insurance). The different nations of the U.K. (England, Scotland, Wales, and Northern Ireland) have different organisational systems, but in England (which accounts for over 80% of the U.K. population) funding for healthcare mostly flows from the Department of Health and Social Care, through NHS England, to regional clinical commissioning groups (CCGs) on a capitation basis, and then onto hospitals and other providers on the basis of activity following a national tariff. The National Institute for Health and Care Excellence (NICE) is currently assessing the effectiveness and cost-effectiveness of such testing, but this paper presents an independent economic evaluation not connected to the NICE assessment.

A number of studies have shown that reflex testing of colorectal cancer cases for Lynch syndrome is effective and also cost-effective [4,5,6,7,8]. Demonstrating cost-effectiveness for reflex testing is important to enable testing to be covered/reimbursed. More recent evidence has emerged that reflex testing of endometrial cancer cases for Lynch syndrome may also be cost-effective [9,10,11,12,13], but much of this evidence is limited in its scope, for example, most have not included the option of not testing for Lynch syndrome as a comparator and have only compared testing strategies [9,10,11,12]. We recently published a model-based economic evaluation using prevalence and diagnostic accuracy data estimated from published literature [13], but this cannot adequately address that different testing strategies may not only have different performance characteristics, but may also identify different Lynch syndrome cases and face different challenges in a heterogeneous population. For example, MSI testing may both be less specific in older patients (where somatic *MLH1* hypermethylation is known to be more common in colorectal cancer [14]) and may be less sensitive to identify path_MSH6 and path_PMS2 cases (i.e., pathogenic variants in *MSH6* and *PMS2*) [15].

The Proportion of Endometrial Tumours Associated with Lynch Syndrome (PETALS) study [16] was conducted in a large U.K. gynaecological cancer centre to evaluate the effectiveness of reflex testing in endometrial cancer cases and to estimate the prevalence of Lynch syndrome in this population. A total 500 endometrial cancer cases were tested with IHC and MSI (300 recruited prospectively, 200 identified retrospectively having been consented for research), with *MLH1* methylation testing and germline next generation sequencing (NGS) being conducted where indicated. Sixteen women were diagnosed with Lynch syndrome.

For an economic evaluation of reflex testing for Lynch syndrome in endometrial cancer patients to be most valuable for decision makers, it should use reliable, consistent, and unbiased evidence for key diagnostic performance parameters, and it should account for the differing values of identifying Lynch syndrome cases according to their age and genotype.

## 2. Methods

We performed an economic evaluation on the basis of patient-level data from the PETALS study [16]. For each participant in PETALS, we simulated their diagnostic outcomes (and associated costs) according to different diagnostic strategies. We used a Markov model to extrapolate the expected lifetime costs and health outcomes for each participant on the basis of their diagnostic outcome. These were combined, along with estimated costs and benefits of cascade testing for Lynch syndrome in relatives of confirmed cases, to estimate the expected lifetime costs and quality-adjusted life years (QALYs) for each diagnostic strategy, as well as to perform a cost-effectiveness analysis (see Table 1) to determine the optimal strategy.

### 2.1. Selection of Interventions and Comparators

#### 2.1.1. The Manchester Approach

The Manchester approach uses a four-MMR protein immunohistochemistry panel to triage all endometrial cancer cases. Where no assays indicate loss of expression, the case is discharged from further testing for Lynch syndrome. If the tumour shows loss of MLH1 and/or PMS2 (but MSH2 and MSH6 are expressed), *MLH1* methylation testing is conducted on DNA from tumour cells and from normal adjacent tissue. Cases showing hypermethylation in tumour cells are discharged from further testing for Lynch syndrome. If the tumour shows loss of MSH2 and/or MSH6, or if the tumour shows loss of MLH1 and/or PMS2 expression unexplained by *MLH1* methylation, a NGS panel is used to identify constitutional pathogenic variants in the MMR genes (and *EPCAM*).

Informed consent is obtained in the gynaecology clinic prior to NGS testing, but only patients in whom a pathogenic MMR variant is found are referred for genetic counselling.

#### 2.1.2. Comparator Strategies

**Figure 1:**
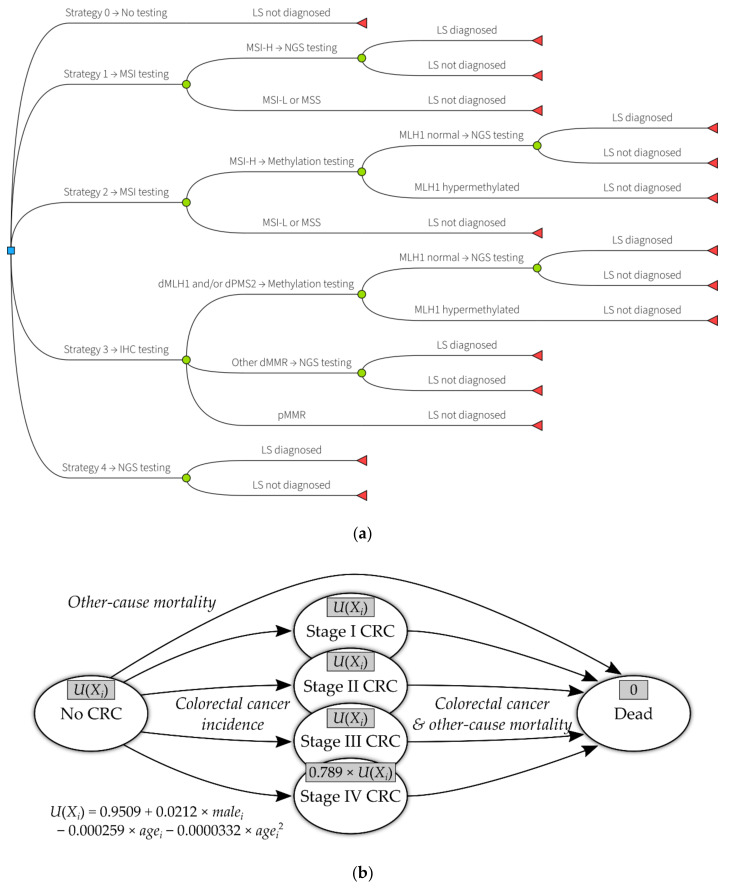
Model diagrams: (**a**) interventions and comparators in the economic evaluation; LS: Lynch syndrome; MSI: microsatellite instability; MSI-H: microsatellite instability—high; MSI-L; microsatellite instability—low; NGS: next generation sequencing; MSS: microsatellite stable; IHC: immunohistochemistry; dMLH1: deficient MLH1 expression; dPMS2: deficient PMS2 expression; dMMR: mismatch repair deficiency; pMMR: proficient mismatch repair. (**b**) Markov model structure (self-links not shown for clarity); grey boxes in each health state give the utility value (QALY (quality-adjusted life years) weight) for that state; U(X_i_): utility value for individual *I* with characteristics *X_i_*; CRC: colorectal cancer.

The principal comparators to the Manchester approach (which is labelled strategy 3) that were included in this evaluation are (see Figure 1a):

- No testing for Lynch syndrome in endometrial cancer cases (strategy 0);
- Triage by microsatellite instability testing, followed by NGS (strategy 1);
- Triage by microsatellite instability testing, then *MLH1* methylation testing, followed by NGS (strategy 2);
- Direct NGS (strategy 4).

Microsatellite instability testing typically includes a panel of five markers, and instability at two or more of these is classed as microsatellite instability—high (MSI-H). Instability at a single marker is referred to as microsatellite instability—low (MSI-L). Instability in zero markers is referred to as microsatellite stable (MSS). MSI is frequently caused by *MLH1* promoter hypermethylation, but the MSI markers have no diagnostic utility for recognising this, and thus either all MSI cases must undergo methylation testing or none.

In addition to these comparators, we explored certain variations in scenario analyses:

- Using a two-MMR protein IHC panel (including only MSH6 and PMS2 antibodies);
- Treating MSI-L as indicative for further Lynch syndrome testing;
- Using clinical criteria (age or PREMM₅ (PREdiction Model for gene Mutations, 5-gene version) score [17]) to select patients at higher risk of having Lynch syndrome.

### 2.2. Economic Evaluation Approach

#### 2.2.1. Diagnostic Testing

For each participant in the PETALS study, we simulated their diagnostic pathway according to each diagnostic strategy. For example, if the tumour of a patient in PETALS had absent MSH6 but was MSS, they would be simulated as discharged from further testing in strategies 1 and 2, even though in PETALS they would have received further testing. Where *MLH1* methylation testing was not ordered in PETALS (on the basis of IHC results), it was assumed the test would not have shown hypermethylation. Patients not undergoing NGS in PETALS were assumed not to have Lynch syndrome.

When an endometrial cancer patient (proband) is diagnosed with Lynch syndrome, cascade testing of their relatives is simulated. For each proband, we modelled on average six relatives having an appointment with a general practitioner in relation to Lynch syndrome, of whom 4.7 attended genetic counselling, 3.3 underwent predictive genetic testing, and 1.5 were diagnosed with Lynch syndrome [13]. The relatives were modelled irrespective of the true Lynch syndrome status and diagnostic outcome for the proband, so that there was a consistent population across interventions.

#### 2.2.2. Extrapolation

A Markov model was used to extrapolate future costs and health outcomes (measured in QALYs). The Markov model was adapted from the model used by Snowsill et al. [13]. It was ported from Excel to R [18] and built using the heemod package [19].

The Markov model included colorectal cancer as a downstream consequence of having Lynch syndrome (diagnosed or undiagnosed) and colonoscopy as a risk-reducing intervention for those with diagnosed Lynch syndrome. The incidence of colorectal cancer is modelled on the basis of which (if any) MMR gene contains a pathogenic variant and the age of the individual [13]. For relatives, the sex also influences the incidence of colorectal cancer [13]. Colonoscopy has been assumed to directly reduce the incidence of colorectal cancer [8,20] and to make detection of colorectal cancer in earlier stages more likely [8,21]. Colorectal cancer survival was modelled in terms of stage, with stage IV having the worst prognosis and stage I the best. A model diagram is shown in Figure 1b.

The Markov model does not incorporate other Lynch syndrome-associated cancers, such as future gynaecological cancer, cancers of the non-colorectal digestive tract, and cancers of the biliary and urinary tracts [1]. It also does not include other preventative measures such as gynaecological surveillance and aspirin chemoprevention, as these are not routinely used in clinical practice.

### 2.3. Methods for Estimating Costs

Costs are presented in 2016/2017 pounds Sterling (£; GBP), inflated using the Hospital and Community Health Services Pay and Prices Index [22] to 2015/2016 and then by 1.1% to 2016/2017.

#### 2.3.1. Diagnostic Costs

The microcosting conducted by Ryan et al. [23] provided estimates for the majority of diagnostic costs, however, we adjusted the unit costs to add on costs for staff time (as the original microcosting included only staff salaries). This involved scaling labour costs by a factor of 2.08, and represented additional costs of employers’ pension contributions, employers’ National Insurance Contributions, and overheads (management, administrative and estates staff, non-staff overheads), but did not include capital overheads. This was estimated by taking the average ratio of total cost (excluding capital overheads) to salary for hospital-based scientific and professional staff in bands 4 to 7 [22].

We assumed that IHC with a two-MMR antibody panel would cost half the cost of the standard test. This likely represents a best case scenario as there are likely to be some economies of scale. It was assumed that filtering cases on the basis of age would not incur any cost. The cost of calculating, recording, and communicating the PREMM₅ score for a patient was assumed to be £3.58 (on the basis of requiring 5 min of gynaecology registrar time) [22].

Costs for genetic counselling were estimated on the basis of Slade et al. [24], with post-test counselling costing £133 (for both probands and relatives) and pre-test genetic counselling costing £172 for relatives. The Manchester approach follows the mainstreaming cancer genetics paradigm of not offering pre-test genetic counselling for probands [25]. As a scenario analysis, we assumed counselling costs of £428 for the first visit (post-test counselling for probands, pre-test counselling for relatives) and £403 for a follow-up visit (post-test counselling for relatives) [26].

A summary of diagnostic costs is given in Table 2.

#### 2.3.2. Long-Term Costs

The Markov model used to extrapolate long-term costs and outcomes includes costs for colonoscopic surveillance and costs associated with colorectal cancer. Colonoscopies were assumed to cost £583 [27], whereas the cost of colorectal cancer was dependent on the age of the patient and stage of cancer at time of diagnosis, ranging from £807 (aged 80+, stage IV) to £14,490 (aged 40–49, stage III) [28].

### 2.4. Methods for Estimating Health Benefits

#### 2.4.1. Lynch Syndrome Cases Identified

For strategy 0 (no testing), we assumed no Lynch syndrome cases are identified. For strategy 1 (MSI triage), we assumed a true Lynch syndrome case would have been correctly identified if the tumour demonstrated MSI-H. For strategy 2 (MSI and *MLH1* methylation triage), we assumed a true Lynch syndrome case would have been correctly identified if the tumour demonstrated MSI-H and if methylation testing showed no hypermethylation or was not conducted in the PETALS study (as this would only be the case if IHC showed normal MLH1 expression). For strategy 3 (IHC and *MLH1* methylation triage; the Manchester approach) we assumed a true Lynch syndrome case would have been correctly identified if the tumour demonstrated complete (i.e., not patchy) absence of 1+ MMR proteins, and that *MLH1* methylation testing showed no hypermethylation or was not indicated. For strategy 4 (direct NGS), we assumed that all true Lynch syndrome cases would be identified.

#### 2.4.2. Long-Term QALYs

The Markov model used to extrapolate long-term costs and outcomes includes a baseline profile for health-related quality of life that declines with age [29], as well as a loss of quality of life for those with stage IV colorectal cancer (utility is scaled by 0.789, on the basis of a systematic review and meta-regression [30]).

### 2.5. Methods for Handling Uncertainty and Heterogeneity

Our analyses naturally address heterogeneity in the patient population by projecting future costs and outcomes for each participant in the PETALS trial individually on the basis of their age and MMR genotype (sporadic/wildtype, path_MLH1, path_MSH2, path_MSH6, path_PMS2). We additionally modelled a heterogeneous group of relatives by dividing a cohort of 310 relatives from the Wales regional genetics service (personal communication, Dr Ian Frayling, Cardiff University, Cardiff, Wales, U.K., 2012) into age quintiles, calculating the mean age for each of these (24.5, 35.7, 45.0, 53.2, and 66.8 years of age, respectively) and simulating outcomes for men and women, starting at each of these ages with each of the MMR genotypes (50 unique combinations).

Uncertainty is principally handled through a combined non-parametric bootstrap and probabilistic sensitivity analysis approach. We performed 200 iterations, and in each of these iterations, the PETALS population was resampled with replacement and the model was re-run for the resampled population using values for each parameter sampled from probability distributions.

We explored a number of important scenario analyses to reflect alternative strategies for service provision or key parameter assumptions. We explored whether it is cost-effective to apply clinical criteria prior to testing, either restricting testing to those diagnosed with endometrial cancer under the age of 70 or to those with a calculated PREMM₅ score ≥ 2.5%. We also explored the cost-effectiveness of testing under a “worst case” scenario, where the effectiveness of colonoscopic surveillance was estimated from an Italian study [31] that showed the worst performance for surveillance in a systematic review [32], and it was interpreted in the least favourable light to produce a hazard ratio for colorectal cancer incidence of 0.929 (compared to a base case assumption of 0.387 [8,20]).

Our deterministic analyses utilised the full PETALS population and central estimates for parameter values (and so incorporated heterogeneity but not parameter uncertainty), whereas our probabilistic analyses incorporated heterogeneity and parameter uncertainty, as described above.

### 2.6. Further Details of Economic Evaluation

Table A1 gives further details of the inputs for the economic evaluation.

## 3. Results

### 3.1. Lynch Syndrome Cases Identified

As reported elsewhere [16], 16 Lynch Syndrome cases were identified amongst the 500 endometrial cancer patients. These patients were younger than those with sporadic endometrial cancer—the mean age for Lynch syndrome cases was 54 years versus 63.5 years for sporadic cases; 15 of 16 Lynch syndrome cases were aged under 70 years versus 314 of 484 sporadic cases. Path_MSH6 variants accounted for eight cases, whereas there were four path_MSH2 variants and two each of path_MLH1 and path_PMS2. Detailed family history was available for 299 patients and allowed calculation of PREMM₅ score [17]; 11 of 13 Lynch syndrome cases with detailed family history had PREMM₅ ≥ 2.5%, as did 153 of 286 (53%) sporadic cases.

### 3.2. Cost per Lynch Syndrome Case Identified

In the PETALS study there were 16 cases of Lynch syndrome identified. Strategy 0 (no testing) would not be expected to identify any of these, but would incur no cost. Strategies 1 and 2 (MSI-based strategies) would be expected to identify nine of these, whereas strategy 3 (the Manchester approach) and strategy 4 (direct NGS) would be expected to identify all of them. The Manchester approach was the least expensive testing strategy and identified the most cases (tied with direct NGS, which was the most expensive strategy). The MSI-based strategies were dominated by (more expensive and less effective than) the Manchester approach and therefore not predicted to be cost-effective. The Manchester approach was estimated to incur costs of £1700 per case of Lynch syndrome identified (i.e., an ICER of £1700 per Lynch syndrome (LS) case identified). Table 3 shows the costs and cases identified for each strategy.

In the combined bootstrap and probabilistic sensitivity analysis, we found that the 95% credible interval for the ICER of the Manchester approach versus no testing was £1117 to £3275 per LS case identified, and that the Manchester approach dominated the other testing strategies with a probability of 0.475.

When limiting to endometrial cancer cases aged < 70 years, the Manchester approach identified 15 Lynch syndrome cases (100% of those aged < 70 years). Strategy 2 (MSI and *MLH1* methylation testing triage) was the cheapest strategy, but only identified nine (60%) Lynch syndrome cases, and was extendedly dominated by (more expensive and less effective than a combination of) the Manchester approach and strategy 0 (no testing). The Manchester approach costed £1300 per Lynch syndrome case identified if testing only those aged < 70 years. When comparing universal reflex testing with the Manchester approach to testing only those aged < 70 years, universal testing costed £7900 per additional Lynch syndrome case detected.

When applying PREMM₅ risk prediction and only testing those with a score ≥ 2.5%, the Manchester approach identified 11 (100%) Lynch syndrome cases. Strategy 2 (MSI and *MLH1* methylation testing triage) was the cheapest strategy but only identified eight (73%) Lynch syndrome cases, and was extendedly dominated by the Manchester approach and no testing. The Manchester approach costed £1100 per Lynch syndrome case identified when PREMM₅ risk scoring was incorporated. When comparing universal reflex testing with the Manchester approach to testing with PREMM₅ risk scoring, universal testing costed £2100 per additional Lynch syndrome case detected.

We found that IHC with a two-antibody (MSH6 and PMS2) panel would detect all 16 (100%) Lynch syndrome cases in the PETALS study, and on the basis of the assumption that it would halve the cost of IHC testing, it would also result in cost saving, costing £1200 per Lynch syndrome case identified.

Including MSI-L as a “test positive” in strategy 2 led to the identification of one additional Lynch syndrome case and the requirement for further unnecessary testing in 20 patients, resulting in an additional cost of £5600 per Lynch syndrome case identified versus only treating MSI-H as a “test positive”. The Manchester approach still dominated MSI-based testing strategies.

### 3.3. Impact of Surveillance on Life Expectancy, Colorectal Cancer Outcomes, and QALYs

The Markov model predicts that identifying Lynch syndrome and putting surveillance in place improves life expectancy, but the gain is dependent on which MMR gene is affected and the age of the individual. For example, a 34-year-old proband with path_MLH1 would be expected to gain 4.3 years in life expectancy, whereas a 69-year-old proband with path_PMS2 would only be expected to gain 0.4 years in life expectancy. The average life expectancy gain would be expected to be 1.8 years amongst the probands with Lynch syndrome who are diagnosed and receive surveillance. The average gain in life expectancy from surveillance for female relatives with Lynch syndrome was found to be 4.0 years, whereas it was found to be 3.5 years for male relatives.

The Markov model predicted that for the 16 women diagnosed with Lynch syndrome in PETALS, surveillance would reduce the expected lifetime number of colorectal cancers from 6.3 to 3.2. This included an increase in the number of stage I colorectal cancers from 1.2 to 2.2, and a reduction in the number of colorectal cancers diagnosed in later stages (stage II: 3.1 to 0.4; stage III: 1.3 to 0.4; stage IV: 0.7 to 0.3).

Surveillance was also predicted to improve lifetime QALYs (note that lifetime QALYs are discounted at 3.5% per year, unlike life expectancy). Surveillance was expected to lead to a gain of 0.6 QALYs on average amongst probands with Lynch syndrome, but this ranged from 0.05 to 1.38 QALYs, depending on age and genotype. The average gain in QALYs from surveillance for relatives with Lynch syndrome was found to be 1.1 QALYs for women and 1.0 QALYs for men, with younger relatives and those with path_MLH1 and path_MSH2 variants benefiting most.

### 3.4. Cost per QALY

When diagnostic outcomes were extrapolated into lifetime costs and health outcomes (measured in QALYs) and combined with diagnostic costs, we found that strategies 1 and 4 were not cost-effective. When we fixed model parameters at their central estimates, we predicted that the Manchester approach would be cost-effective (see Table 3). When we considered only the endometrial cancer patients themselves, the Manchester approach incurred on average £100 additional cost and generated an average 0.020 QALYs, giving an ICER of £5000 per QALY gained. This gain in QALYs would be inconsequential (it corresponds to around 1 week in perfect health), but it was concentrated in the minority of patients who had Lynch syndrome, who gained, on average, 0.624 QALYs from the Manchester approach. When we also included cascade testing of relatives, the Manchester approach incurred, on average,e £286 additional cost per endometrial cancer patient and had an ICER of £5500 per QALY (compared to strategy 2, which had an ICER of £3800 per QALY).

**Figure 2:**
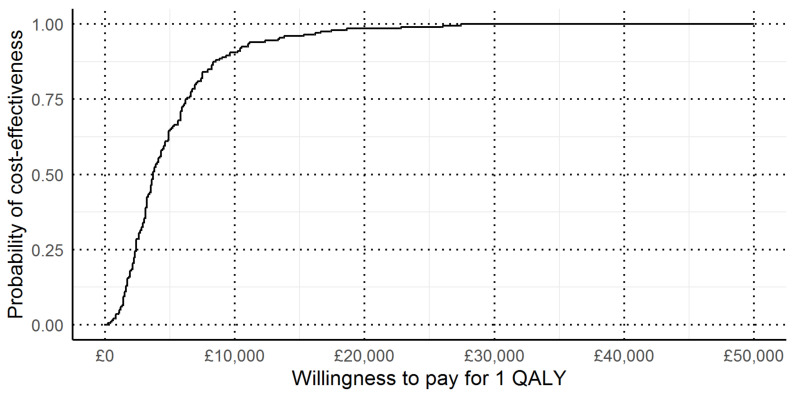
Cost-effectiveness acceptability curve for the Manchester approach versus no testing. This figure shows, on the basis of how much a decision maker is willing to pay for 1 QALY, what the probability is that the Manchester approach is cost-effective compared to no testing.

In the combined bootstrap and probabilistic sensitivity analysis, we found that the 95% credible interval for the ICER of the Manchester approach versus no testing was £700 to £17,000 per QALY gained (see Figure 2).

When testing was restricted to those aged < 70 years, the Manchester approach and strategy 2 (MSI and *MLH1* testing triage) could be cost-effective, depending on the cost-effectiveness threshold. The Manchester approach produced a greater health benefit at a cost of £5500 per QALY, and was therefore cost-effective at a threshold of £20,000 per QALY. Comparing universal testing to only testing those aged < 70 years, universal testing was cost-effective, costing an additional £12,900 per QALY gained.

When PREMM_5_ risk scoring was included (with a threshold of 2.5%), we found that the Manchester approach was cost-effective, producing the greatest health benefit at an additional cost of £6000 per QALY gained relative to strategy 2 (the next best option). Universal testing was found to be cost-effective compared to PREMM₅ testing, costing an additional £11,700 per QALY gained.

When the effectiveness of colonoscopy was estimated from Arrigoni et al. 2005 [31], the cost-effectiveness of testing worsened, as expected. Strategy 2 was found to be cost-effective at a threshold of £20,000 per QALY, whereas the Manchester approach was cost-effective with a threshold of £30,000 per QALY. The Lynch syndrome cases identified by the Manchester approach but not strategy 2 were mainly path_MSH6 cases (5/7) who had a lower risk of colorectal cancer than path_MLH1 and path_MSH2 cases [33,34], and therefore they and their relatives already benefit less in absolute terms from surveillance.

Using IHC with a two-antibody (MSH6 and PMS2) panel would (assuming that no Lynch syndrome cases were missed) save approximately £15 per patient compared to using the full panel with no change in QALYs.

Including MSI-L as a “test positive” would cost £9500 per QALY gained compared to only considering MSI-H a “test positive”, but would be extendedly dominated by the Manchester approach, and thus would not be cost-effective.

When the cost of genetic counselling was estimated from NHS reference costs for consultant-led clinical genetics outpatient appointments, the Manchester approach remained cost-effective, costing £6700 per QALY gained. The total average lifetime costs per participant (including any costs for relatives) were increased by £60 over the base case where genetic counselling costs were taken from a costing study for *BRCA* mutations.

## 4. Discussion

Our study suggests that the Manchester approach is cost-effective compared to no testing and compared to other reflex testing strategies, costing around £5500 per QALY gained, and further that universal testing (without restricting by age or predicting risk of Lynch syndrome using PREMM₅) is cost-effective. We found that restricting IHC testing to a two-antibody (MSH6 and PMS2) panel would improve cost-effectiveness, but this finding was based on no Lynch syndrome cases failing to display deficient expression for one of these proteins within PETALS, whereas other studies have shown that a two-antibody panel is inferior to a four-antibody panel [35]. We found that including MSI-L as a “test positive” result was not sufficient to make MSI-based testing cost-effective compared to the Manchester approach.

The only current published study of reflex testing for Lynch syndrome in women with endometrial cancer to include no testing as a comparator found that IHC and *MLH1* methylation triage was cost-effective, but costed approximately £14,000 per QALY gained for a 60-year-old endometrial cancer patient, and was not cost-effective for patients aged 65 years and older [13]. The current study based its extrapolation of long-term costs and QALYs on that study, and thus differences in cost-effectiveness were driven by alternative assumptions regarding diagnostic testing, key amongst which are (i) lower costs of IHC (and MSI and *MLH1* methylation testing) on the basis of a microcosting study [23] rather than estimates from pathologists and laboratory price lists, and (ii) not including pre-test genetic counselling costs for probands.

This study has a number of strengths. As it was based on a single, large, high-quality trial, it captured all of the associations between patient characteristics and tumour test results, such as the relationship between age and *MLH1* hypermethylation, and the possibility of differential test performance for pathogenic variants in different MMR genes. This compares favourably to existing modelling studies that have had to make assumptions about the performance across heterogeneous groups [11,12,13]. The PETALS study itself has strengths, such as including a U.K. mixed metropolitan population and being conducted in a non-insurance based system, meaning that there were minimal barriers to participants consenting to germline testing [36]. The costs associated with diagnostic testing were measured contemporaneously [23] in the same hospital, which contributed to high internal validity.

The study also has some limitations that should be considered. PETALS identified 16 participants with Lynch syndrome, but there were no cases detectable by MSI but not IHC. Such cases are hypothetically possible and their absence in PETALS means that this possibility was not explored through bootstrapping. Importantly, NGS was not conducted on all PETALS participants (although it was conducted even if tumour tests did not demonstrate MMR deficiency in young patients and those with suggestive family histories), and it was possible that a direct NGS strategy may have identified additional pathogenic MMR variants. Further, as PETALS was a research study, participants were consented for NGS upon entering the study, rather than being consented only after tumour triage testing; it was possible that consenting for NGS at a different time-point could have resulted in lower uptake.

A significant proportion of the value of identifying Lynch syndrome was to implement colonoscopic surveillance to reduce the incidence and mortality due to colorectal cancer, however, the effectiveness of colonoscopic surveillance has not been evaluated in an experimental setting. The Manchester approach is more able to detect path_MSH6 cases (and likely more able to detect path_PMS2 cases) than MSI-based testing, but these women are also at lower risk of colorectal cancer, and therefore if colonoscopic surveillance is less effective than generally thought, testing for these cases and providing surveillance may not be cost-effective.

Our model to extrapolate long-term costs and benefits focusses on colorectal consequences of Lynch syndrome, and does not model future gynaecological cancer or gynaecological surveillance in relatives, nor does it model the cost or effect of aspirin (which may be used to reduce the incidence of Lynch syndrome associated cancers [37]), nor does it model extra-colonic non-gynaecological cancers.

Although the methods employed in this study are broadly applicable, it is important to note that there are frequently challenges to generalising the results of cost-effectiveness analyses to other settings [38].

## Figures and Tables

**Table 1:** Economic evaluation characteristics.

Decision Problem	What is the Relative Cost-Effectiveness of Strategies to Identify Lynch Syndrome in Women with Endometrial Cancer
Interventions and comparators	Strategy 0: No testing Strategy 1: MSI triage followed by NGS Strategy 2: MSI and *MLH1* methylation testing triage followed by NGS Strategy 3: IHC and *MLH1* methylation testing triage followed by NGS (the Manchester approach) Strategy 4: Direct NGS
Type of economic evaluation, costs, and health outcomes	Cost-effectiveness analysis: Diagnostic costs and Lynch syndrome cases identified (no cost-effectiveness threshold identified) Cost-utility analysis: Lifetime costs and QALYs for women with endometrial cancer and their relatives (cost-effectiveness threshold £20,000 per QALY)
Model type	Decision tree and Markov model implemented in R
Key data source	PETALS study (diagnostic accuracy study conducted in Manchester)
Perspective	NHS and PSS, costs in pounds sterling (£; GBP) in 2016/17 prices
Time horizon	Lifetime
Discounting	3.5% for costs and QALYs
Analysis of uncertainty	Non-parametric bootstrap resampling of participants in a clinical study and parametric sampling of model parameters (probabilistic sensitivity analysis)

MSI: microsatellite instability; IHC: immunohistochemistry; NGS: next generation sequencing; QALY: quality-adjusted life years; PETALS: The Proportion of Endometrial Tumours Associated with Lynch Syndrome; NHS: National Health Service; PSS: personal social services; GBP: (Great British) Pounds sterling (£).

**Table 2:** Diagnostic testing unit costs.

Item	Unit Cost (£, GBP)	Source
Calculate PREMM₅ score	3.58	PSSRU 2017 [22]
MMR IHC (4 protein panel)	30.36 ¹	Ryan et al. 2019 [23]
MMR IHC (2 protein panel)	15.18	Assumed half cost of 4 protein panel
MSI testing	36.63 ¹	Ryan et al. 2019 [23]
*MLH1* methylation testing (strategy 2)	22.84 ¹	Ryan et al. 2019 [23]
*MLH1* methylation testing (strategy 3)	32.65 ¹	Ryan et al. 2019 [23]
Obtain consent for NGS	13.64 ¹	Ryan et al. 2019 [23]
NGS	236.35 ¹	Ryan et al. 2019 [23]
Post-test genetic counselling (probands)	133.15	Slade et al. 2016 [24]
Pre-test genetic counselling (relatives)	171.73	Slade et al. 2016 [24]
Predictive genetic testing (relatives)	166.32	Slade et al. 2016 [24]
Post-test genetic counselling (relatives)	133.15	Slade et al. 2016 [24]

**Key:** IHC, immunohistochemistry; MMR, mismatch repair; MSI, microsatellite instability; NGS, next generation sequencing; PREMM_5_: PREdiction Model for gene Mutations, 5-gene version. **Notes:** ¹ Labour costs scaled by 2.08 to include additional costs other than salary.

**Table 3:** Deterministic base case cost-effectiveness analysis.

Strategy	Costs (£)	Effectiveness Outcome	ICER
Short-term	Diagnostic pathway costs ¹	Lynch syndrome cases identified ¹	Additional diagnostic pathway cost per Lynch syndrome case identified
Strategy 0 No testing	0	0	—
Strategy 1 MSI	41,512	9	Dominated
Strategy 2 MSI and *MLH1* methylation	27,523	9	Dominated
Strategy 3 IHC and *MLH1* methylation	27,183	16	1699
Strategy 4 Direct NGS	127,125	16	Dominated
Lifetime	Lifetime costs (proband/proband and relatives)	Lifetime QALYs (proband/proband and relatives)	Additional cost per QALY gained (proband only/proband and relatives)
Strategy 0 No testing	120 642	7.64 104.61	— —
Strategy 1 MSI	223 842	7.65 104.65	Dominated Dominated
Strategy 2 MSI and *MLH1* methylation	195 815	7.65 104.65	Extendedly dominated 3738
Strategy 3 IHC and *MLH1* methylation	220 928	7.66 104.67	5003 5459
Strategy 4 Direct NGS	419 1128	7.66 104.67	Dominated Dominated

Key: ICER, incremental cost-effectiveness ratio; IHC, immunohistochemistry; MSI, microsatellite instability; NGS, next generation sequencing; QALY, quality-adjusted life year. Notes: ¹ Calculated on the basis of PETALS population (500 endometrial cancer patients). Strategies are arranged in ascending order of effectiveness. ICERs were calculated versus the next most effective strategy on the cost-effectiveness frontier.

## Appendix A

**Table A1:** Details of model parameters.

Parameter	Base Case Value	Uncertainty (Distribution, 95% CI) ¹
**Population Characteristics**		
Prevalence of Lynch syndrome	3.20%	Bootstrap, 1.96% to 5.68%
Prevalence of *path_MLH1*	0.40%	Bootstrap, 0.03% to 1.41%
Prevalence of *path_MSH2*	0.80%	Bootstrap, 0.24% to 2.03%
Prevalence of *path_MSH6*	1.60%	Bootstrap, 0.68% to 3.19%
Prevalence of *path_PMS2*	0.40%	Bootstrap, 0.02% to 1.33%
Age of Lynch syndrome cases (years)	54	Bootstrap, 47.0 to 60.9
Age of *path_MLH1* cases	40.5	Bootstrap, 31.4 to 50.2
Age of *path_MSH2* cases	53.8	Bootstrap, 44.7 to 62.3
Age of *path_MSH6* cases	59.1	Bootstrap, 49.5 to 67.8
Age of *path_PMS2* cases	47.5	Bootstrap, 16.6 to 79.5
Age of sporadic cases	63.5	Bootstrap, 62.4 to 64.6
Relatives per proband	6	Gamma, 1.80 to 11.96
Probability relative accepts counselling	77.70%	Beta mixture, 73.8% to 81.1%
Probability relative accepts testing after counselling	71.60%	Ratio, 67.4% to 76.4%
Probability relative has Lynch syndrome	44.00%	Beta, 41.0% to 47.3%
Probability relative is female	52.80%	Beta, 47.6% to 57.0%
**Diagnostic Effectiveness**		
Strategy 1 sensitivity	0.563	Bootstrap, 0.256 to 0.818
Strategy 1 specificity	0.835	Bootstrap, 0.804 to 0.868
Strategy 2 sensitivity	0.563	Bootstrap, 0.256 to 0.818
Strategy 2 specificity	0.967	Bootstrap, 0.948 to 0.981
Strategy 3 sensitivity	1	Bootstrap, 1.000 to 1.000
Strategy 3 specificity	0.967	Bootstrap, 0.954 to 0.986
**Disease Natural History**		
Colorectal cancer risk for proband aged 60 years to age 80 years ²^,^³		
*path_MLH1*	39.50%	Log-normal model, 33.5% to 47.4%
*path_MSH2*	35.70%	Log-normal model, 28.9% to 42.1%
*path_MSH6*	19.90%	Log-normal model, 12.1% to 28.1%
*path_PMS2*	10.20%	Log-normal model, 1.3% to 30.2%
Sporadic	2.19%	Not varied
Colorectal cancer risk for female relative aged 60 years to age 80 years ²^,^³		
*path_MLH1*	30.80%	Log-normal model, 24.1% to 40.1%
*path_MSH2*	27.00%	Log-normal model, 20.5% to 36.3%
*path_MSH6*	12.80%	Log-normal model, 6.9% to 22.7%
*path_PMS2*	5.50%	Log-normal model, 0.5% to 24.9%
Sporadic	2.19%	Not varied
Colorectal cancer risk for male relative aged 60 years to age 80 years ²^,^³		
*path_MLH1*	35.20%	Log-normal model, 28.7% to 43.8%
*path_MSH2*	31.40%	Log-normal model, 23.8% to 40.7%
*path_MSH6*	16.30%	Log-normal model, 9.5% to 26.2%
*path_PMS2*	7.70%	Log-normal model, 0.9% to 30.7%
Sporadic	3.48%	Not varied
*5-year endometrial cancer mortality risk for probands* ³		
Lynch syndrome cases	10.40%	Exponential model, 6.1% to 15.2%
Sporadic cases (x = age at diagnosis)		
x < 45	12.40%	Exponential model, 10.5% to 14.2%
45 ≤ x < 55	13.10%	Exponential model, 11.9% to 14.5%
55 ≤ x < 65	14.50%	Exponential model, 13.6% to 15.4%
65 ≤ x < 75	21.50%	Exponential model, 20.7% to 22.5%
x ≥ 75	36.90%	Exponential model, 35.3% to 38.9%
*10-year other-cause mortality risk* ³		
Woman aged 60	7.60%	Not varied
Woman aged 70	20.30%	Not varied
Man aged 60	11.40%	Not varied
Man aged 70	28.10%	Not varied
*5-year colorectal cancer mortality* ³		
Lynch syndrome case, by stage		
Stage I	4.50%	Exponential model, 2.1% to 8.0%
Stage II	15.80%	Exponential model, 7.7% to 27.0%
Stage III	38.60%	Exponential model, 20.8% to 59.2%
Stage IV	93.40%	Exponential model, 92.9% to 93.9%
Sporadic case, by stage		
Stage I	6.80%	Exponential model, 6.1% to 7.5%
Stage II	22.90%	Exponential model, 22.5% to 23.6%
Stage III	52.30%	Exponential model, 51.7% to 53.0%
Stage IV	93.40%	Exponential model, 92.9% to 93.9%
**Colonoscopic Surveillance Effectiveness**		
Hazard ratio for incidence of colorectal cancer from colonoscopic surveillance	0.387	Log-normal model, 0.164 to 0.753
*Colorectal cancer stage distribution*		
Lynch syndrome case, under surveillance		
Stage I	68.60%	Dirichlet distribution, 52.9% to 81.8%
Stage II	10.50%	Dirichlet distribution, 3.3% to 22.3%
Stage III	12.80%	Dirichlet distribution, 5.0% to 23.4%
Stage IV	8.10%	Dirichlet distribution, 1.8% to 18.8%
Lynch syndrome case, not under surveillance		
Stage I	18.80%	Dirichlet distribution, 9.2% to 34.2%
Stage II	48.80%	Dirichlet distribution, 31.7% to 63.9%
Stage III	21.20%	Dirichlet distribution, 10.7% to 32.8%
Stage IV	11.20%	Dirichlet distribution, 4.1% to 22.8%
Sporadic case		
Stage I	17.60%	Dirichlet distribution, 17.2% to 18.0%
Stage II	27.00%	Dirichlet distribution, 26.5% to 27.5%
Stage III	29.50%	Dirichlet distribution, 29.1% to 30.0%
Stage IV	25.90%	Dirichlet distribution, 25.4% to 26.4%
**Utility Values**		
*Baseline utility by gender and age (years)*		
Woman, 40	0.887	Regression model, 0.826 to 0.949
Woman, 50	0.855	Regression model, 0.766 to 0.944
Woman, 60	0.816	Regression model, 0.696 to 0.937
Woman, 70	0.77	Regression model, 0.611 to 0.931
Woman, 80	0.718	Regression model, 0.513 to 0.924
Woman, 90	0.659	Regression model, 0.403 to 0.917
Man, 40	0.909	Regression model, 0.845 to 0.974
Man, 50	0.876	Regression model, 0.787 to 0.968
Man, 60	0.837	Regression model, 0.717 to 0.963
Man, 70	0.791	Regression model, 0.630 to 0.954
Man, 80	0.739	Regression model, 0.534 to 0.945
Man, 90	0.68	Regression model, 0.424 to 0.938
*Utility modifiers (multipliers)*		
Stage IV colorectal cancer	0.789	Beta, 0.721 to 0.844
**Costs**		
*Diagnostic costs*		
IHC	£30.36	Gamma, £21.10 to £44.95
MSI	£36.63	Gamma, £23.90 to £55.35
MLH1 methylation post-IHC	£32.65	Gamma, £20.96 to £44.72
MLH1 methylation post-MSI	£22.84	Gamma, £14.53 to £35.86
NGS	£236.35	Gamma, £152.85 to £326.52
Post-test counselling (proband)	£133.15	Gamma, £83.00 to £185.05
Consent to test (proband)	£13.64	Gamma, £12.60 to £14.79
GP referral (relative)	£36.40	Gamma, £24.16 to £48.46
Pre-test counselling (relative)	£171.73	Gamma, £110.07 to £252.03
Predictive mutation testing (relative)	£166.32	Gamma, £116.27 to £245.42
Post-test counselling (relative)	£133.15	Gamma, £84.75 to £183.80
PREMM_5_ scoring	£3.58	Gamma, £2.35 to £5.29
*Surveillance costs*		
Colonoscopy	£583.34	Gamma, £383.84 to £809.88
Interval between colonoscopies	2.1 years	Log-normal, 1.54 to 2.89 years
*Colorectal cancer costs (lifetime discounted costs, by stage and age)*		
Stage I, <50	£8754	Gamma, £5907 to £11,966
Stage I, 50–59	£5712	Gamma, £3793 to £7993
Stage I, 60–69	£4623	Gamma, £2990 to £6602
Stage I, 70–79	£3178	Gamma, £2125 to £4701
Stage I, ≥80	£1380	Gamma, £917 to £1868
Stage II, <50	£8741	Gamma, £5529 to £12,172
Stage II, 50–59	£7016	Gamma, £4367 to £10,348
Stage II, 60–69	£5352	Gamma, £3447 to £7751
Stage II, 70–79	£3455	Gamma, £2166 to £5124
Stage II, ≥80	£1546	Gamma, £923 to £2216
Stage III, <50	£14,490	Gamma, £8742 to £21,370
Stage III, 50–59	£9692	Gamma, £6269 to £13,227
Stage III, 60–69	£7259	Gamma, £4500 to £10,369
Stage III, 70–79	£4485	Gamma, £2965 to £6123
Stage III, ≥80	£1561	Gamma, £1044 to £2424
Stage IV, <50	£11,705	Gamma, £7719 to £17,169
Stage IV, 50–59	£8444	Gamma, £5762 to £11,688
Stage IV, 60–69	£6509	Gamma, £4461 to £9038
Stage IV, 70–79	£4365	Gamma, £2636 to £5946
Stage IV, ≥80	£807	Gamma, £514 to £1097

¹ Bootstrap 95% confidence intervals calculated using the normal approximation on a suitable transformed scale (logit was preferred for proportions but arcsine was used when bootstrap iterations included 0 or 1). ² Estimates for those with Lynch syndrome are in the presence of colonoscopic surveillance. ³ Calculations conducted absent competing risks.
